# Diagnosing eyewitness identifications with reaction time‑based Concealed Information Test: the effect of viewpoint congruency between test and encoding

**DOI:** 10.1007/s00426-023-01857-1

**Published:** 2023-07-21

**Authors:** Melanie Sauerland, Linda Geven, Astrid Bastiaens, Bruno Verschuere

**Affiliations:** 1 Department of Clinical Psychological Science, Section Forensic Psychology, Faculty of Psychology and Neuroscience, Maastricht University, P.O. Box 616, 6200 MD Maastricht, The Netherlands; 2 Institute for Criminal Law and Criminology, Faculty of Law, Leiden University, Leiden, The Netherlands; 3 Department of Clinical Psychology, University of Amsterdam, Amsterdam, The Netherlands

## Abstract

Mistaken eyewitness identifications continue to be a major contributor to miscarriages of justice. Previous experiments have suggested that implicit identification procedures such as the Concealed Information Test (CIT) might be a promising alternative to classic lineups when encoding conditions during the crime were favorable. We tested this idea by manipulating view congruency (frontal vs. profile view) between encoding and test. Participants witnessed a videotaped mock theft that showed the thief and victim almost exclusively from frontal or profile view. At test, viewing angle was either congruent or incongruent with the view during encoding. We tested eyewitness identification with the RT-CIT (*N* = 74), and with a traditional simultaneous photo lineup (*N* = 97). The CIT showed strong capacity to diagnose face recognition (*d* = 0.91 [0.64; 1.18]), but unexpectedly, view congruency did not moderate this effect. View congruency moderated lineup performance for one of the two lineups. Following these unexpected findings, we conducted a replication with a stronger congruency manipulation and larger sample size. CIT (*N* = 156) showed moderate capacity to diagnose face recognition (*d* = 0.63 [0.46; 0.80]) and now view congruency did moderate the CIT effect. For lineups (*N* = 156), view congruency again moderated performance for one of the two lineups. Capacity for diagnosing face recognition was similar for lineups and RT-CIT in our first comparison, but much stronger for lineups in our second comparison. Future experiments might investigate more conditions that affect performance in lineups vs. the RT-CIT differentially.

## Diagnosing eyewitness identifications with reaction time-based Concealed Information Test: the effect of viewpoint congruency

In 2004, Romano van der Dussen, a Dutchman who lived in Spain, was sentenced to 15½ years in prison on two accounts of assault and rape. The two assault victims and a witness initially did not recognize him from mugshots in the police database. When they saw the mugshots a second time 3 weeks later, they did point out van der Dussen as the perpetrator, and they later identified him again from a police lineup. Apart from the undesirable repeated recognition attempts (Wixted et al., 2021), there were several other issues with the identification procedure in this case. For example, the witness saw the perpetrator from more than 10 m distance, for a very short time, and in the dark, putting her in a very poor condition to identify the perpetrator (Nyman et al., 2019). Another issue concerned the lineup itself: the blond-haired van der Dussen was placed among black-haired foils, making him the only lineup member who matched the perpetrator description (cf. recommendation #4 from Wells et al., 2020). Other warning signs included that no physical evidence linked him to the crimes and that an alibi witness testified that van der Dussen had been at a party 30 km away. In 2004, DNA taken from one of the victims matched with Mark Dixie, a British man convicted for murder. Van der Dussen was only released from prison when Dixie confessed in 2016 (Lindemans, 2019).

Establishing the identity of a perpetrator is at the heart of crime investigation. When investigators have narrowed down their search of a suspect, witnesses may view a live, photo or video lineup that contains the suspect and several foils who are known to be innocent. It is the task of the eyewitness to identify the person who they saw commit the crime—or to reject the lineup if that person is not in the lineup. Decades of research on eyewitness memory have identified conditions that support and impede eyewitnesses in making accurate identification decisions and have resulted in policy changes that are aimed at supporting eyewitness memory. Yet, as the van der Dussen case demonstrates, improper lineup procedures still happen in practice, putting innocent suspects at risk of misidentification and conviction (e.g., Christianson et al., 1992; Davies & Griffiths, 2008; Epifanio v. Madrid, 2009; Garrett, 2011; Thompson-Cannino et al., 2009; van Koppen & van der Horst, 2006; Wagenaar, 2009). Under such circumstances, error rates for lineups can be high, with an average of about 50% across conditions (e.g., Clark et al., 2008; Fitzgerald & Price, 2015). As a result, the use of explicit identification procedures has decreased considerably in some countries (e.g., the Netherlands) and other countries dismiss them altogether (e.g., South Korea, Indonesia). Indirect assessments of recognition, such as the Concealed Information Test (CIT; Lykken, 1959) might provide for an alternative. Advantages of indirect measures of recognition include that they are less intentional, faster, and more stimulusvdriven than direct measures of recognition. But it is important to map their boundary conditions (Verschuere & Meijer, 2014). Here, we tested the validity of the CIT as a means of diagnosing face recognition under viewing conditions that were congruent or incongruent during encoding and testing.

The CIT is a well-established memory detection technique (Lykken, 1959; for a review see Verschuere et al., 2011) that resembles lineups in some aspects. Similar to a lineup, a CIT includes different types of stimuli: the correct, crime-related stimulus (e.g., murder weapon: a pistol) that is embedded in several plausible stimuli that are not crime-related (e.g., a rifle, a knife, an axe, an injection needle). Instead of relying on explicit responses (“This is the murder weapon”), the CIT infers explicit recognition in an indirect way, namely from neural (e.g., blood oxygen level-dependent response in fMRI; P300 event-related potential), physiological (e.g., skin conductance reaction), or behavioral (e.g., reaction times) responses. In our example, police could ask the suspect about the murder weapon: Was it …. A rifle? … An axe? …A knife? … A pistol? … An injection needle? Differential reactions to the actual murder weapon, the pistol, compared to other stimuli, indicate recognition. When combining multiple questions, for example about stolen goods, the crime scene, and the location of the crime, the CIT can detect recognition with high validity (Meijer et al., 2014, 2016).

A variation of the classic CIT, the reaction time-based CIT (RT-CIT) requires only a single computer and enables web-based testing with high reliability and validity (Kleinberg & Verschuere, 2015; for a theoretical analysis, see Verschuere & De Houwer, 2011). The RT-CIT uses reaction times to index recognition of concealed information. To assure attention to the stimuli and avoid mindless and indifferent responses to all stimuli, the RT-CIT introduced a third type of stimuli, namely targets.^1^*Targets* are non-crime-related stimuli that the participants need to detect, and usually study just before the test. During the RT-CIT task, the stimuli appear on screen sequentially and participants press one key for the targets and another for all other stimuli. Building on the example above, participants may learn that the CIT will examine recognition of the murder weapon and to press the YES key whenever encountering the target (e.g., a rifle) and the NO key for all other stimuli. For innocent (unknowledgeable) participants, all NO reaction times should be similar. For guilty (knowledgeable) participants, the option *pistol* should stand out and affect their response times. Longer reaction times for NO responses to the crime-related stimulus than NO responses to irrelevants provide an index of recognition. A meta-analysis reported a large effect size of Cohen’s *d* = 1.04 (corrected), confirming the diagnosticity of the RT-CIT (Suchotzki et al., 2017).

In the first application of the CIT in the context of face recognition, participants made explicit identifications in an event-related potential-based CIT after they watched four mock crimes (Lefebvre et al., 2007). Both the CIT and explicit identifications revealed recognition of the perpetrator. Yet, the electrophysiological index of recognition may have been the result of the simultaneous explicit identification. In recent, stricter applications of the CIT protocol in a typical eyewitness paradigm, the RT-CIT showed a good capacity to differentiate the stimulus film actors (i.e., probes) from irrelevants in three experiments (*d* = 1.21; Georgiadou et al., 2019, Experiment 2b; *d*s = 0.85 and 0.74; Sauerland et al., 2023) and moderate capacity in another (*d* = 0.39; Sauerland et al., 2019, Experiment 4). Additionally, one experiment included a probe-absent CIT condition and demonstrated a good capacity of the CIT to differentiate between guilty and innocent suspects (Sauerland et al., 2023, Experiment 2).

Not all attempts of applying RT-CIT for diagnosing face recognition were successful, however. A series of five experiments reported a small average effect size (*d* = 0.14; Sauerland et al., 2019). These conflicting findings within facial recognition RT-CIT experiments might originate from differences in encoding conditions and event complexity. Experiments with moderate to large effects included only two rather than four actors and provided ample close-ups of both (Georgiadou et al., 2019, Experiment 2b; Sauerland et al., 2019, Experiment 4, Sauerland et al., 2023). In the experiment with the largest effect size (Georgiadou et al., 2019, Experiment 2b), encoding was additionally enhanced by presenting the pictures of the actors for 15 s after participants had viewed the stimulus film and prior to taking the RT-CIT. From an applied eyewitness identification perspective, this setup was somewhat flawed, though, because the presented picture was identical to the picture used in the CIT (Burton, 2013). Nevertheless, these experiments combined suggest that a certain degree of memory strength might be required to ensure reliable performance in the CIT. Although encoding conditions are not under the control of investigators, this finding might be useful in cases with good encoding conditions.

In the current experiment, we manipulated the congruency of viewing angle at encoding vs. testing to further investigate the impact of encoding conditions on the validity of the RT-CIT as an index of facial recognition. For half of our participants, the viewing angle at encoding and testing matched (both frontal or both profile view), for the other half, encoding and testing viewing angle mismatched (encoding: frontal—testing: profile and vice versa). Recognition of unfamiliar faces becomes more difficult as angular rotation between encoding and recognition increases. Face recognition experiments have first demonstrated this effect with research designs that used photos both at encoding and testing (Crookes & Robbins, 2014; Johnston & Edmonds, 2009; Liu & Chaudhuri, 2002). Recently, an eyewitness identification paradigm where participants viewed a filmed mock theft at encoding and a photo lineup at testing confirmed this effect (Colloff et al., 2021). Altogether, these findings suggest that we store unfamiliar faces in a viewpoint-dependent manner.

Congruency of stimuli at encoding and testing can also affect the size of the CIT effect. In one experiment, participants encoded stimulus items either verbally or pictorial (van der Cruyssen et al., 2021). The subsequent RT-CIT protocol presented both types of modalities. Confirming the idea of a modality-match advantage, the CIT effect was larger when the modalities at encoding and retrieval matched (*ds* between 0.40 and 0.60) than when they mismatched (*ds* between −0.14 and 0.59). Another experiment tested the effect of encoding–testing congruency by varying the level of abstraction of the presented stimuli (Geven et al., 2019). Participants viewed either exemplar (e.g., Mercedes) or categorical stimulus items (e.g., car) at encoding and the CIT protocol matched or mismatched this stimulus representation. Again, congruent stimulus presentation at encoding and testing elicited a stronger CIT effect (*ds* = 0.47 and 0.55) than incongruent stimulus presentation (*ds* = −0.23 and 0.06). Another set of two experiments tested whether angular rotations of the crime-related images in the CIT protocol, compared to encoding, affected the CIT effect (Hsu et al., 2020). A CIT effect emerged in all conditions, but decreased for more occluding angles such as 90° and 270°. Combined, these findings further support the idea of a superiority performance of matched conditions across different tests of recognition. However, previous work has not tested the effect of view congruency of face stimuli on the strength of the CIT effect.

In the current line of research, participants viewed a stimulus film that showed one actor primarily from the front and one actor primarily in profile view. At test, participants completed an RT-CIT (Experiments 1 and 3) or made lineup decisions (Experiments 2 and 4). The lineup data served as a benchmark of eyewitness performance. Participants viewed the facial stimuli at test from the same as or a different perspective than at encoding. We expected better identification and hence a stronger CIT effect (i.e., difference in reaction times to probes vs. irrelevants) when viewing angle was congruent, compared to incongruent (CIT congruency effect; hypothesis 1). We also predicted that identification performance in lineups would vary as a function of congruency (lineup congruency effect; hypothesis 2). The relative capacity of the CIT and lineups to diagnose face recognition is of strong applied interest, but we had no hypothesis for this comparison. Experiments 1 and 2 did not confirm our hypotheses and showed largely inconclusive results. We therefore conducted two preregistered replication experiments (Experiments 3: RT-CIT; and 4: lineup) for which we strengthened the view congruency manipulation and increased power.

## Method

The experiments received ethical approval by the Ethics Review Committee of the faculty (approval codes 231_140_12_2020_S3 and OZL_231_140_12_2020_S9). The Inquisit scripts and data are available here: https://osf.io/bru5w/. One of the films can be viewed here: https://mediasite.maastrichtuniversity.nl/Mediasite/Play/98a09c905ca04df9a7771e4c03bd57e31d. We cannot share all the pictures because we do not have permission of the depicted individuals. We did not preregister Experiments 1 and 2. The preregistration for Experiments 3 and 4 is here: https://osf.io/3sryq.

### Participants

#### Power analyses

We based the power analysis with G*Power (Faul et al., 2007, 2009) on the comparison between CIT and lineup performance because the power of this comparison is weakest. For Experiments 1 and 2, we entered *df* = 1, *α* = .05, power = .80, and *φ* = .30 for a chi-square test, to be able to detect at least moderate differences between the two identification procedures. The required sample size for each of the two comparisons was 88 per comparison and 176 in total (i.e., comparing both identification procedures for congruent vs. incongruent viewing conditions).

Accounting for 15% drop out or exclusions, we planned to test 202 participants in total, with 101 respondents in each Experiment 1 and 2. We were able to test the planned number of participants for the lineup experiment, but fell somewhat short of this for the CIT experiment. Yet, we still had strong power (1 − *β* = .90) to detect a small interaction effect (*f* = .15) between CIT effects and view congruency, and strong power (1 − *β* > .99) to detect a moderate interaction effect (*f* = .25). However, we may have been underpowered to detect a difference in recognition performance in the CIT vs. lineup if the effect size were moderate (1 − *β*  = .76 to detect a moderate effect; 1 − *β*  = .99 to detect a large effect; see Discussion).

For Experiments 3 and 4, we entered *df* = 1, *α* = .05, power = .95, and *φ* = .30 for a chi-square test to increase power. The required sample size for each of the two comparisons was 145 and 290 in total (i.e., comparing both identification procedures for congruent vs. incongruent viewing conditions). Accounting for 15% drop out or exclusions, we planned to test 332 participants in total, with 166 respondents in each Experiment 3 and 4.

#### ***Samples***

For Experiments 1 and 2, we recruited the participants via social media platforms such as Facebook, the university’s SONA research participation system, and respondent seeking platforms such as SurveyCircle (http://www.surveycircle.com) and SurveySwap (http://www.surveyswap.io). On these platforms, researchers collect points for participating in other people’s study, and receive a higher ranking for their own study in return. Participants received 0.5 study credits if they participated via SONA or one of respondents seeking platforms.

In Experiment 1 (CIT), we excluded 4 of 88 participants because they did not pass the attention check (see materials) and 10 because they made too many errors (i.e., > 50%, cf. Kleinberg & Verschuere, 2015) in response to target trials in the CIT condition. The remaining 74 participants (29 men, 45 women, age 18–58; *M*_age_ = 27.41, *SD*_age_ = 7.32) were master’s students (39.2%), bachelor’s students, (32.4%), or non-students (28.4%). Student participants studied at the faculties of Arts and Social Sciences (14.9%), Psychology and Neuroscience (14.9%), Health, Medicine and Life Sciences (8.1%), Law (6.8%), or Science and Engineering (2.7%). Participants’ mother tongues were English (41.9%), German (16.2%), Dutch (13.5%), Norwegian (2.7%), or other (25.7%).

In Experiment 2 (lineups), we excluded 4 of 101 participants because they did not pass the attention check. The remaining 97 participants (34 men, 62 women, 1 non-binary, age 16–77, *M*_age_ = 27.78, *SD*_age_ = 11.63) were bachelor’s (55.7%) and master’s students (30.9%) at the faculties of Psychology and Neuroscience (37.1%), Arts and Social Sciences (11.3%), Science and Engineering (7.2%), Health, Medicine and Life Sciences (4.1%), or Law (1.0%). About one in eight participants were non-students (13.4%). Participants’ mother tongues were German (41.2%), English (28.9%), Dutch (19.6%), or other (10.3%).

For Experiments 3 and 4, we recruited participants via the platform prolific. Preselection inclusion criteria were speaking English fluently, Caucasian ethnicity to prevent other-group bias as much as possible (Meissner & Brigham, 2001), and being between 18 and 50 years old to avoid confounding age effects (Brackmann et al., 2019; Fitzgerald & Price, 2015; Martschuk & Sporer, 2018). Participants received £7.50/h as reimbursement.

In Experiment 3 (CIT), from the initial *n* = 166, we excluded six participants because their data were incomplete and four participants because they did not pass the attention check. One participant did the whole procedure twice, and, while we had not anticipated this possibility and it is therefore a deviation from our preregistration, we excluded the second participation. There were no exclusions for having less than 50% accuracy on any of the three CIT trial types. The final 156 participants (88 men, 65 women, 2 preferred not to say, 1 missing, age 19–49, *M*_age_ = 26.48, *SD*_age_ = 6.85) worked full-time (35.9%) were unemployed (19.9%), worked part-time (12.8%), did not provide data about employment (10.9%), or were not in paid work (1.9%). Half of the participants were students (53.2%) and 10.3% did not provide data about their student status. Participants’ nationality varied between 20 countries in Europe, North America, Africa, and Asia. Countries with more than two participants included Poland (30.1%), Portugal (23.1%), Greece (8.3%), Italy (7.7%), Spain (7.1%), Hungary (6.4%), UK (3.8%), Slovenia (2.6%), and South Africa (1.9%).

In Experiment 4 (lineups), of 166 participants, we excluded 5 participants because their data were incomplete and 5 because they did not pass the attention check. Six participants participated twice. As in Experiment 3, we excluded the second participation. The final 156 participants (91 men, 64 women, 1 preferred not to say, age 19–50, *M*_age_ = 30.21, *SD*_age_ = 7.88) worked full-time (44.2%) or part-time (16.6%), did not provide data about employment (16.0%), were unemployed (10.8%), or not in paid work (5.2%). One-third of participants were students (32.0%) and 11.6% did not provide data about their student status. Participants’ nationality varied between 25 countries in Europe, North America, Africa, and Asia. Countries with more than two participants included Portugal (34.6%), Poland (18.3%), Italy (17.3%), Greece (14.4%), UK (11.5%), Spain (7.7%), Czech Republic, Latvia, South Africa (5.8% each), Hungary (4.8%), Netherlands, and Slovenia (3.9% each).

### Design

In the CIT Experiments 1 and 3, we used a within-subjects 2 (view congruency at encoding vs. test: congruent [frontal-frontal or profile-profile] vs. incongruent [frontal-profile or profile-frontal]) × 2 (stimulus type: probe vs. irrelevant) design to test the effect of view congruency on identification performance in a CIT. The two actors served as probes. The dependent measures were the reaction times to probes and irrelevants in each condition.

In the lineup Experiments 2 and 4, we manipulated view congruency in a one-factorial between subjects design with two levels (view congruency: congruent vs. incongruent) to test the effect of view congruency on identification accuracy in an actor-present lineup. We coded identification decisions as accurate (hits) or inaccurate (foil selections, false rejections).

In all experiments, participants viewed one probe during encoding in frontal view and one probe in profile view. At test, all images were either all in frontal or all in profile view. We counterbalanced the role of the two probes across participants: for 50% of the participants, probe A was the thief and probe B was the victim and for the other 50% it was the other way round.

### Materials

#### Stimulus films

We created four versions of a stimulus film. All film versions depicted the theft of a handbag, and they all showed the same action. In each film, which lasted 68 or 69 s, the thief was primarily visible from a frontal view and the victim from a profile view or vice versa. The roles of the two female actors (thief or victim) were counterbalanced across viewing angle conditions. Participants viewed the films without audio.

The action can be described as follows: on a square in a pedestrian mall, the future thief asks the future victim for directions. The thief then heads off in the pointed direction. In the next shot, the victim is sitting on a bench looking at her phone, with her handbag next to her. The thief sneaks up from behind, grabs the handbag and runs away. Table 1 shows an overview of facial frontal and profile viewing time across the different film versions. To strengthen the congruency manipulation in Experiments 3 and 4, we added coverage that was in line with the conditions and cut coverage that was not. Viewing times of close-up and distant shots were adjusted to be more similar for both the thief and victim role across the different films. Additionally, if we could not cut coverage that did not fit the condition, we darkened the film for that section, resulting in more consistent frontal or profile views than in Experiments 1 and 2.

**Table 1:** Facial frontal and profile viewing time in four stimulus films (in s) in Experiments 1 and 2 vs. Experiments 3 and 4

Film version	Role	Actor	View	Frontal close-up	Frontal distant	Profile close-up	Profile distant	Overall facial view	Overall duration film
*Experiments 1 and 2*									
Film 1	Thief	A	Frontal	15	6	2	8	31	69
	Victim	B	Profile	0	8	15	18	41	
Film 2	Thief	A	Profile	0	3	16	9	28	68
	Victim	B	Frontal	25	13	2	1	41	
Film 3	Thief	B	Frontal	13	4	1	10	28	68
	Victim	A	Profile	0	6	15	12	33	
Film 4	Thief	B	Profile	0	0	18	10	28	68
	Victim	A	Frontal	25	5	2	6	38	
*Experiments 3 and 4*									
Film 1	Thief	A	Frontal	19	1	0	0	20	59
	Victim	B	Profile	3	0	19	4	26	
Film 2	Thief	A	Profile	18	2	0	3	23	63
	Victim	B	Frontal	0	0	18	9	27	
Film 3	Thief	B	Frontal	0	1	21	2	24	59
	Victim	A	Profile	18	4	2	0	24	
Film 4	Thief	B	Profile	0	0	20	3	23	56
	Victim	A	Frontal	19	3	2	2	26	

#### CIT and lineup photos

We used the same facial photographs for the CIT task and lineups. Photographs showed probes, targets, and irrelevants from the front or in 90° profile from the collarbone up, without jewelry, eyeglasses, or hair accessories and with loose hair. All clothing was edited to be black and the probes wore different clothing in the photograph than in the film. To avoid recognition of one probe by a small mole on the cheek, we edited the target and irrelevant pictures corresponding to this probe to include a mole as well.

The pictures fitted the general description of the probes depicted in the different stimulus events, as determined by presenting independent samples of mock witnesses (*n*s between 25 and 26) who had not viewed the stimulus event with a description of each probe (or probe replacement) together with five fillers (e.g., ‘She is about 20–22 years old. She has blonde-red and wavy hair. She has a slim to normal figure.’). These mock witnesses then selected the person from the lineup who matched the description best (Doob & Kirshenbaum, 1973). If all fillers are good alternatives to the probe, each lineup member should receive an approximately equal number of selections from the mock witnesses. The effective lineup size gives an indication in how far this is the case. Ideally, the effective lineup size should be close to its nominal size—six in our case. The effective lineup size Tredoux’s E ranged from 4.3 to 4.6 (of a possible 6), thereby marking them a fair picture selection (Tredoux, 1998, 1999).

#### Reaction time‑based Concealed Information Test (Experiments 1 and 3)

We presented the CIT protocol, using Inquisit 6.4.2 and 6.6.1 web player, respectively. The software recorded reaction times in milliseconds. All stimuli pictures were 388 × 462 pixels. We used one combined CIT protocol for the thief and the victim, with the images for thief and victim intermixed. Depending on the condition, the images in the CIT were either all displayed in frontal view or all in profile view. Participants received instructions to place their index fingers on the L and A key for the duration of the experiment and to press the L key as fast as possible in response to a facial stimulus, with the exception of the two targets. For these stimuli, they should press the A key rather than the L key. Participants viewed the targets for 30 s, accompanied by instructions to encode these faces.

In Experiment 1, participants went through a practice block showing each of the stimuli (probes, targets, fillers) once. Participants received feedback if their response was incorrect or too slow (*wrong*, or *too slow*). They had 1500 ms to react before the next stimulus was shown following an inter-stimulus interval of 250, 500, or 700 ms to prevent strategic slowing (Suchotzki et al., 2021). The “too slow” feedback appeared after 800 ms, but the responses were recorded up to 1500 ms. Participants completed a second practice block if they had more than 50% errors or misses on target responses in the first practice block or a mean response time longer than 800 ms. After a second practice block, participants continued with the actual task regardless of performance. Prior to the actual task, participants viewed the target faces for five more seconds and received a reminder of how to respond.

To improve web-based reaction time responding, we included a stepwise practice phases (Kleinberg & Verschuere, 2015) in Experiment 3. Participants initially saw the targets for 25 s, accompanied by instructions to encode these faces. In the first practice block, participants responded without a time limit and received feedback about accuracy (*wrong*). After this block, participants saw the target once more for 5 s. In the second practice block, the stimuli additionally disappeared after 1500 ms. In the final practice block, we increased time pressure by adding *too slow* feedback. During each practice block, participants saw every CIT stimulus twice (i.e., 24 trials). If participants made more than two errors in practice block 2 or 3, the block was repeated. Prior to the start of the actual task, participants saw the targets for another 5 s. Thus, the total viewing time of the targets was the same in Experiments 1 and 3 (i.e., 35 s).

During the actual task, every stimulus appeared 21 times, in random sequence. The CIT stimuli consisted of 2 * 6 pictures (2 probes, 2 * 4 fillers, 2 targets), resulting in 12 * 21 = 252 trials in total. The question “Do you recognize this person?” appeared above every stimulus and the labels “YES” and “NO” on the left and right sides. If participants pressed the wrong key or reacted too slowly, they received feedback (*wrong*, *too slow*).

*Follow-up photo display* Participants in Experiments 1 and 3 viewed a photo recognition display after the CIT ask. The display included 14 pictures: the 12 pictures used in the CIT and a thief and victim replacement. The view of these photos was congruent with the view during encoding. Participants indicated the women they (explicitly) recognized from the stimulus film at the very end of the experiment. This allowed us to roughly determine if participants in the CIT conditions had explicit memory of the probes. In both Experiments 1 and 3, a binomial test against 1/7 odds (chance level of 0.143) showed that participants identified the thief (*M*_*E1*_ = 0.58, [0.47; 0.70]; *M*_*E3*_ = 0.36, [0.0.28; 0.44]) and the victim (*M*_*E1*_ = 0.61, [0.49; 0.72]; *M*_*E3*_ = 0.43, [0.35; 0.51]) above chance level from the photo display, *ps* < 0.001. Recognition accuracy in this task^2^ did not systematically differ as a function of view congruency time, with *BF*_*01*_ = 1.49 for both thief and victim in Experiment 1 and *BF*_*01*_ = 5.27 for the thief and 4.55 for the victim in Experiment 3.

#### Lineups (experiments 2 and 4)

We composed separate actor-present thief and victim frontal and profile lineups with six photographs each. Lineups included the probe (i.e., guilty suspect), the four irrelevants, and the target. Lineup members were numbered 1–6, with the numbers arranged in two rows of three pictures (i.e., a simultaneous lineup). The position of the probe in the lineup varied between two, three, and four for one probe and between three, four, and five for the other probe.

Participants read that “police are trying to identify the thief from the film you just saw. Because you saw the theft, they present you with a lineup. Note that the thief may or may not be present in this lineup. If you are not sure or don't know, you can select the "not present" option.” For the victim lineup, the instructions were as follows: “You will now view a lineup referring to the victim. Note that the victim may or may not be present in this lineup. If you are not sure or don't know, you can select the "not present" option.” Following their identification decision, participants indicated how confident they were about their identification decision on a scale from 0 to 100% after each lineup. We have not analyzed or reported the confidence data. The sequence of the lineups was fixed (thief-victim), but thief and victim actors were counterbalanced.

#### Attention check

Participants answered three attention check questions, namely, two multiple-choice questions with five response options (Where did the actors first meet? What color was the stolen handbag? What was the victim doing when the handbag was stolen?) In Experiment 1, 75 participants answered all 3 items correctly (1 error: *n* = 9; 2 errors: *n* = 4). In Experiment 2, 97 participants answered all three items correctly and four participants answered one item correctly. In Experiment 3, 143 participants answered all 3 items correctly (1 error: *n* = 13; 2 errors: *n* = 3; 3 errors: *n* = 1). In Experiment 4, 146 participants answered all 3 items correctly (1 error: *n* = 10; 2 errors: *n* = 3; 3 errors: *n* = 2).

### Procedures

Testing occurred online, using Qualtrics (Experiment 1 and 2) and milliseconds/Inquisit (all experiments). Participants received instructions to use a PC or laptop, but not a phone or tablet, in a quiet space without disruptions. In all experiments, after providing consent, participants were randomly assigned to one of the four conditions. Participants were instructed to pay attention to every detail. To insert a short retention interval, participants provided demographic information (Experiments 1 and 2 only) and answered the three attention check items after watching the video. In Experiments 1 and 3, participants then worked on the CIT task and the follow-up photo display; in Experiments 2 and 4 they viewed the lineups. The testing sessions in Experiments 1 and 3 took approximately 15 min, in Experiments 2 and 4 about 6 min. Following their participation, participants received the debriefing and reimbursement.

### Analyses

For Experiments 1 and 3, using JASP 0.17.1.0, we conducted a within-subjects 2 (view congruency: congruent vs. incongruent) × 2 (stimulus types: probe vs. irrelevants) ANOVA. We included correct reactions only (i.e., excluding behavioral errors^3^) and those that occurred in the time frame between 150 and 1500 ms (following Sauerland et al., 2019). For Experiments 2 and 4, we conducted 2 × 2 chi-square tests to establish the effect of view congruency on identification accuracy. We conducted separate tests for the thief and the victim.

#### Comparison of CIT vs. lineup performance

To compare performance in CIT vs. lineups, we classified CIT performance as accurate or inaccurate, based on an individual effect size (*d*CIT). Following earlier work (Kleinberg & Verschuere, 2015), we used an individual effect size measure for the CIT, i.e., the *d*CIT calculated as [(*M* probe RT − *M* irrelevant RT)/*SD* irrelevant RT]. We classified participants with *d*CIT scores > 0.20 as correct and participants with *d*CIT scores ≤ 0.20 as incorrect. Next, we compared performance (correct vs. incorrect) in the CIT and lineups by means of a 2 × 2 chi-square tests and reported Bayes factors.

## Results

### CIT (Experiments 1 and 3)

#### Experiment 1

**Fig. 1:**
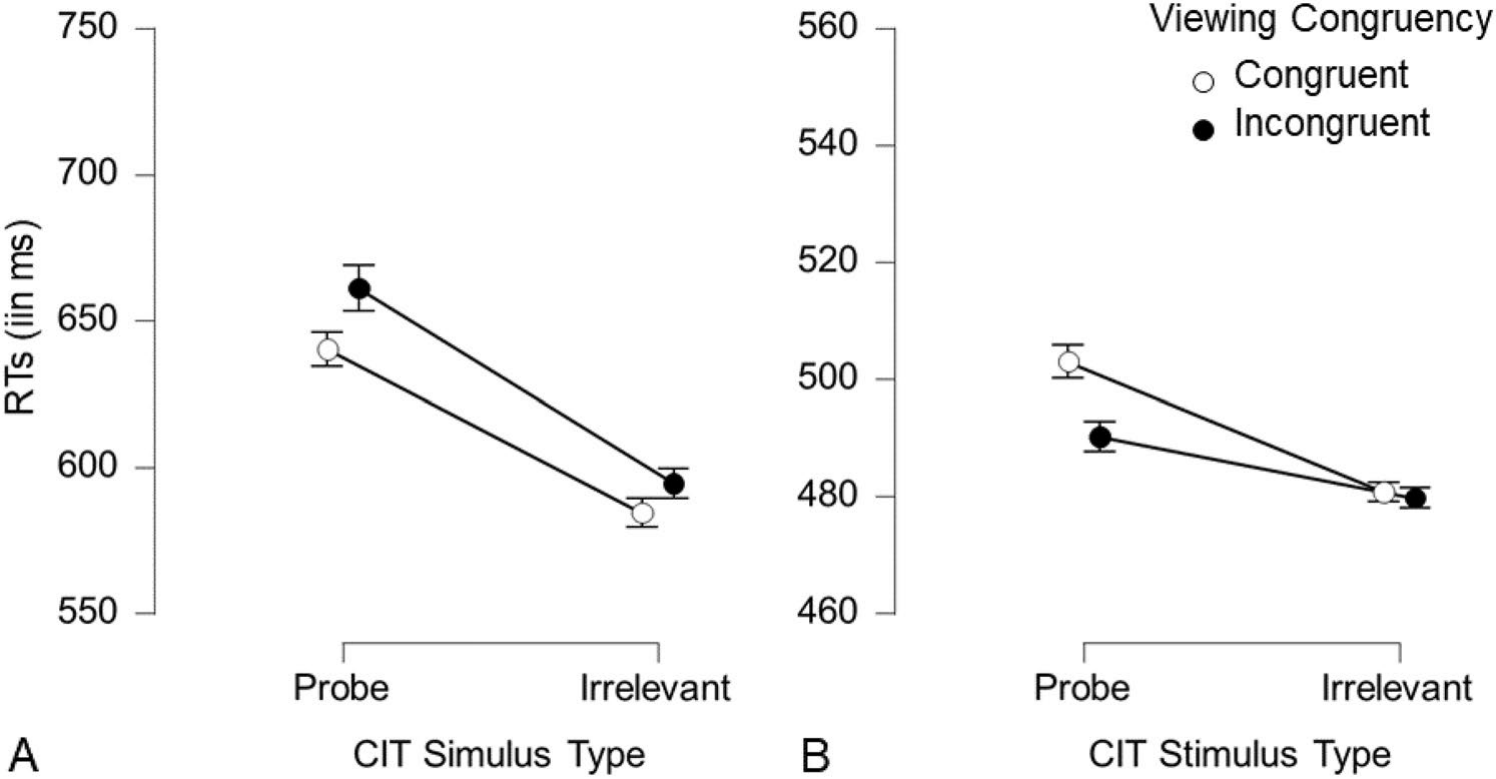
RTs (in ms; with corresponding *SE*s) to probe and irrelevant items in the RT-CIT under congruent vs. incongruent encoding test viewing conditions in Experiment 1 (**A**) and Experiment 3 (**B**)

The main effect of stimulus type was significant, *F*(1, 73) = 61.63, *p* < .001, *d* = 0.91 [0.64; 1.18], with slower responding to probes (*M* = 651 ms; *SD* = 206) than to irrelevants (*M* = 589 ms; *SD* = 160), evidencing a CIT effect. Contrary to hypothesis 1 (CIT congruency effect), the interaction between view congruency and stimulus type was non-significant, as depicted in Fig. 1A, *F*(1, 73) = 1.50, *p* = .224, *η*^2^_p_ = .02, *d* = 0.09.^4^ The probe-irrelevant difference in RTs was large both for the view-incongruent condition, *d* = 0.76 [0.50; 1.02], and the view-congruent condition, *d* = 0.87 [0.60; 1.14].^5^

In an attempt to differentiate absence of evidence from evidence of absence with regard to the non-significant interaction term, we conducted a Bayesian 2 × 2 repeated measures ANOVA with view congruency (congruent vs. incongruent) and stimulus types (probe vs. irrelevants) as within-subjects factors. We used JASP 0.17.1.0 and its default settings (i.e., Cauchy priors with *r* scale = .5) and followed the JASP guidelines (Wagenmakers et al., 2018). Specifically, we added the two main effects to the null model and assessed how likely the data were under the null model (including just the two main effects) versus the alternative model that additionally included the interaction (two main effects + the interaction term). The data spoke against the inclusion of the interaction term *BF*_*M*_ = 0.24.

#### Experiment 3^6^

Experiment 3 again returned a main effect of stimulus type, evidencing a CIT effect, *F*(1, 155) = 63.90, *p* < .001, *d* = 0.65 [0.50; + ∞], with slower responding to probes (*M* = 496 ms; *SD* = 47) than to irrelevants (*M* = 480 ms; *SD* = 43), evidencing a CIT effect, confirming hypothesis 1 (CIT congruency effect). The interaction between view congruency and stimulus type was significant, as depicted in Fig. 1B, *F*(1, 155) = 9.85, *p* = .002, *η*^2^_p_ = .06, *d* = 0.51.^7^ As is clear from Fig. 1B, the RT-CIT effect was larger for the congruent condition than the incongruent condition, *t*(155) = 3.14, *p* = .001, *d* = 0.25 (95% CI: 0.12, + ∞), *BF*_*10*_ = 19.42.

The Bayesian ANOVA also showed that the model with the interaction was the best fitting model, *BF*_*M*_ = 18.51. The probe-irrelevant difference in RTs was small yet significant for the view-incongruent condition, *d* = 0.34 [0.17; + ∞], *BF*_+*0*_ = 660.28, and significant and of moderate size for the view-congruent condition, *d* = 0.59 [0.42; + ∞], *BF*_+*0*_ = 1.57 × 10^9^.

### Lineups (Experiments 2 and 4)

#### Experiment 2

Table 2 shows an overview of identification accuracy rates as a function of view congruency. For the thief, identification accuracy did not differ as a function of view congruency, *χ*^2^(1, *N* = 97) = 0.51, *p* = .474, *φ* = .07. For the victim, identification accuracy did differ as a function of view congruency, *χ*^2^(1, *N* = 97) = 3.99, *p* = .046, *φ* = .20, albeit with a small effect size. A Bayesian chi-square test suggested that the data were inconclusive (*BF*_*10Thief*_ = 0.29; *BF*_*10Victim*_ = 1.76).

**Table 2:** Comparison of correct classification rate of indirect identifications with the RT-CIT and identification accuracy in lineups as a function of view congruency

	Experiment 1—CIT (*N* = 74)	Experiment 2—lineup (*N* = 97)	CIT vs. lineup (Bayes factor)
	Correct classification rate	Identification accuracy	
*Congruent view*	(*n* = 36–38)	(*n* = 43)	*BF*_*01*_
Thief	.63	.67	3.56
Victim	.64	.67	3.84
*Incongruent view*	(*n* = 36–38)	(*n* = 54)	
Thief	.69	.74	3.75
Victim	.63	.47	1.23

	Experiment 3—CIT (*N* = 156)	Experiment 4—lineup (*N* = 156)	CIT vs. lineup (Bayes factor)
	Correct classification rate	Identification accuracy	
*Congruent view*	(*n* = 78)	(*n* = 78)	*BF*_*01*_
Thief	.47	.82	1.60e−4
Victim	.44	.62	.42
*Incongruent view*	(*n* = 78)	(*n* = 78)	
Thief	.37	.65	.01
Victim	.29	.51	.11

*BF*_*01*_ expresses how much more likely the data are under the null hypothesis as compared to the alternative hypothesis of a difference in accuracy of the CIT vs. the lineup

#### Experiment 4

The lower part of Table 2 shows an overview of identification accuracy rates as a function of view congruency. For the thief lineup, identification accuracy differed significantly as a function of view congruency, *χ*^2^(1, *N* = 156) = 5.59, *p* = .018, *φ* = .19, albeit with a small effect size. For the victim lineup, identification accuracy did not differ significantly as a function of view congruency, *χ*^2^(1, *N* = 156) = 1.67, *p* = .196, *φ* = .10. A Bayesian chi-square test confirmed these findings, with anecdotal evidence for the alternative hypothesis for the thief but not the victim lineup (*BF*_*10Thief*_ = 2.80; *BF*_*10Victim*_ = 0.45).

### Identification performance in CIT vs. lineups

Table 2 compares the correct classification rates for the CIT and the Bayes factors for the comparison of the two identification procedures as a function of view congruency. For the comparison of Experiments 1 vs. 2, Bayes factors indicated moderate evidence for the null hypothesis (methods are equivalent). For the comparison between Experiments 3 vs. 4, Bayes factors indicated moderate to very strong or decisive evidence for the alternative hypothesis (methods are not equivalent). Lineup accuracy rates were higher than CIT correct classification rates for all four comparisons.

## Discussion

The RT-CIT is a well-established memory detection technique that allows for indirect assessments of recognition. It might therefore provide a potent alternative to classic lineups as an identification procedure. Here, we tested the validity of the RT-CIT as a tool for diagnosing facial recognition under congruent or incongruent viewing conditions during encoding and testing. We also tested identification performance in a classic lineup condition to create a benchmark of eyewitness performance. Based on the finding that we store unfamiliar faces in a viewpoint-dependent manner (Johnston & Edmonds, 2009), we expected a stronger CIT effect (hypothesis 1) and better lineup performance (hypothesis 2) when viewing angles during encoding and test were congruent, rather than incongruent. Replicating earlier work (Georgiadou et al., 2019; Sauerland et al., 2023), but with entirely different stimulus materials, the RT-CIT showed a good capacity to diagnose face recognition (Experiment 1: *d* = 0.91; Experiment 3: *d* = 0.63). Only Experiment 3 (but not Experiment 1) supported the idea that view congruency moderates this effect (hypothesis 1): the RT-CIT effect was larger for congruent viewing conditions than incongruent viewing conditions. Yet, the effect size for this comparison was small (*d* = 0.25) and may have depended on probe role, as suggested by an exploratory, non-preregistered follow-up analysis. In the two lineup experiments, view congruency moderated lineup performance for one of two lineups, lending only partial support to hypothesis 2. Bayesian analyses suggested that identification performance in the RT-CIT vs. lineups did not differ in our first comparison (Experiment 1 vs. 2), but was much stronger for lineups than the RT-CIT in our second comparison (Experiment 3 vs. 4).

Research in face recognition suggests that people are better at recognizing unfamiliar faces if the viewing angle at test is similar to the viewing angle at encoding (Johnston & Edmonds, 2009). Similarly, although never tested with face stimuli, the diagnosticity of the CIT can vary as a function of congruency of stimuli at encoding and testing (Geven et al., 2019; Hsu et al., 2020; van der Cruyssen et al., 2021). It was therefore unexpected that view congruency did not moderate the CIT effect in Experiment 1. In the replication with a strengthened congruency manipulation and a larger sample, we found a significant interaction effect between the CIT effect and congruency when analyzing both probes together, as preregistered and following the standard procedure in the CIT literature (Suchotzki et al., 2017; Experiment 3). Taken together, the two experiments suggest that view congruency may have a small to moderate effect on the size of the CIT effect and that Experiment 1 may not have had enough power to detect this effect.

Across two experiments, we found only partial support for the hypothesis that view congruency moderates lineup performance (hypothesis 2). Differences in stimulus materials could explain this deviation from the face recognition literature (Johnston & Edmonds, 2009). Experiments in face recognition use photographs both at encoding and at recognition. To simulate the eyewitness situation more closely, we used videos during encoding and photographs during recognition. Despite carefully editing the stimulus films, especially for Experiments 3 and 4, the videos do not show the actors exclusively from a 0° or 90° view but also with slight rotations. Additionally, the richer information about the probes’ appearance during encoding by means of the three-dimensional presentation might counter the effect of view congruency. Future lineup experiments on the effect of view congruency on identification performance might test this idea further.

For being useful in the field, the capacity of the RT-CIT to diagnose face recognition needs to be better or at least equivalent to people’s lineup performance. To compare both methods, we tested identification performance with the RT-CIT and traditional lineups. In our first comparison (Experiment 1 vs. 2), performance in the RT-CIT and lineups was largely equivalent, but in our second comparison (Experiment 3 vs. 4), lineups clearly outperformed RT-CIT. Two previous experiments that compared RT-CIT and lineup performance were inconclusive (Sauerland et al., 2023): some Bayes factors supported the idea that the two procedures were equivalent, some that lineups were superior, and some that RT-CIT was superior. Combined with the current findings, we can only conclude that compelling or consistent evidence for the superiority of one method over the other is still lacking.

## Limitations and future perspectives

One issue of interest is that the CIT effects we observed here – similar to other experiments that tested the validity of the RT-CIT for diagnosing face recognition – was below the average effect size commonly found in RT-CIT experiments (i.e., *d* = 1.04 in a meta-analysis, Suchotzki et al., 2017; cf. Sauerland et al., 2023). Those strong effects in memory detection likely derive from the high self-relevance of the probes and the combination of several stimulus groups in one CIT protocol (e.g., sites of crime, identity of accomplices). Options for enhancing the CIT effect in face recognition – and hence while being limited to facial stimuli – might include the use of familiar targets or increasing the number of targets (cf. Suchotzki et al., 2018). Furthermore, adding different aspects of a person, such as full body pictures with the face covered, clothing, or accessories (Pryke et al., 2004; Sauerland & Sporer, 2008; Sauerland et al., 2013) could be a way of adding more stimulus groups to the CIT protocol.

Another observation on the strength of the CIT effect is that compared to Experiment 1, Experiment 3 elicited faster reaction times, fewer errors, a weaker CIT effect, and poorer recognition performance from the follow-up photo display on a descriptive level. Looking at the differences between those two experiments suggests that the enhanced practice procedure in Experiment 3 may be the cause of these differences. Spreading the encoding of the target faces over three rather than two occasions and increasing the practice blocks from one to two blocks to three to five blocks likely strengthened memory for the targets while at the same time undermining memory for the probes. This seems to have both desirable (low error late) and undesirable effects (weaker CIT effect, weaker recognition performance from the follow-up photo display). Future CIT research should keep such effects of the design of the practice phase in mind when fine-tuning the CIT protocol.

Thus far, comparisons of witness performance in the RT-CIT vs. lineups are inconsistent (the current work; Sauerland et al., 2023). The most relevant question for future investigations could be whether the RT-CIT outperforms lineups under certain conditions, for example whether RT-CIT might be less prone to biases that concern the construction and administration of the procedure than lineups. Because of the indirect character of the RT-CIT, its outcomes might be less vulnerable to the social demands often encountered during lineup administration (cf. Wells & Luus, 1990). Likewise, CIT may benefit people who perform comparably poor in lineups, for example children and older adults (Brackmann et al., 2019; Fitzgerald & Price, 2015; Martschuk & Sporer, 2018). It is also conceivable that encoding conditions differentially affect the two identification procedures. Indeed, in another comparison between RT-CIT and lineups, observation time did not moderate the CIT effect across two experiments, whereas it did moderate the CIT effect in probe-absent lineups in one experiment (Sauerland et al., 2023).

## Conclusion

Mistaken eyewitness identifications continue to be a major contributor to miscarriages of justice. Future research will determine whether there are conditions or target groups for which the RT-CIT is more diagnostic than lineups. Last but not least, an application of the RT-CIT might be beneficial even under the assumption of equivalence when witnesses are reluctant to participate in a lineup, for example, because they fear for their own safety or because they want to protect the perpetrator.

## Data Availability

The Inquisit scripts and data are available here: https://osf.io/bru5w.

## Footnotes

1 Unlike the terminology in the lineup literature, in the CIT, the term *target* describes the person to whom the participant must react differently. The term *probe* describes the person seen during the stimulus event.

2 The purpose of the follow-up photo display was to check that encoding of the probes during the film took place, and it should not be regarded as an additional lineup. There are important differences between the follow-up photo display and a lineup. The follow-up photo display concerns a forced choice task that does not include an option to reject. Furthermore, because the CIT task preceded the follow-up photo display, it should be considered a repeated identification procedure. Recommendations for lineup procedures rightfully advise against repeated identification attempts with the same witness and the same suspect (Wells et al., 2020), because the second identification attempt does not have diagnostic value. This is because the preceding procedure familiarizes participants (witnesses) with the stimuli presented in the photo display and contaminate memory (Wixted et al., 2021). As a result, the accuracy rates for follow-up photo displays can be inflated. On the other hand, based on a focus on target identifications during the CIT protocol, accuracy rates for follow-up photo displays could be impaired.

3 Because error rates are typically very low and lead to less reliable results (Kleinberg & Verschuere, 2015), in line with previous work, we tested our hypotheses exclusively on RTs. For sake of completion: participants in Experiments 1 and 3 made more errors for probes (*M*_*E1*_ = 7.53%; *SD* = 7.88; *M*_*E3*_ = 1.82%; *SD* = 4.32) than for irrelevants (*M*_*E1*_ = 3.20%; *SD* = 5.43; *M*_*E3*_ = 0.84%; *SD* = 1.47), *t*_*E1*_(73) = 5.86, *p* < .001, *d* = 0.68 [0.43; 0.93]; *t*_*E3*_(154) = 2.86, *p* = .005, *d* = 0.23 [0.07; 0. 39].

4 We preregistered to report η^2^_p_ as effect size for interactions. In line with a reviewer’s suggestions, we report Cohen’s *d* as well. The *d* values are based on this calculator: https://www.psychometrica.de/effect_size.html.

5 We also conducted separate analyses for the thief and the victim that were not preregistered. The results were analogous to the collapsed analyses. Specifically, for the thief, the main effect of stimulus type was significant, *F*(1, 72) = 61.00, *p* < .001, *d* = 0.91, while the interaction was non-significant,* F*(1, 72) = 0.24, *p* = .625, *η*^2^_p_ = .003, *d* = 0.11. Likewise, for the victim, the main effect of stimulus type was significant, *F*(1, 72) = 39.98, *p* < .001, *d* = 0.81, while the interaction was not,* F*(1, 72) = 1.85, *p* = .178, *η*^2^_p_ = .025, *d* = 0.32.

6 The analyses of Experiments 3 and 4 are fully in line with the preregistration, with the exception that we preregistered an independent *t* test for Experiment 3, whereas this had to be a paired *t* test.

7 The pattern of results split by probe role (thief vs. victim) was not entirely analogous to the collapsed analyses. Specifically, for the thief, the main effect of stimulus type was significant, *F*(1, 154) = 42.16, *p* < .001, *d* = 0.52, while the interaction was not,* F*(1, 154) = 2.69, *p* = .103, *η*^2^_p_ = .02, *d* = 0.26. For the victim, the main effect of stimulus type was significant, *F*(1, 154) = 27.56, *p* < .001, *d* = 0.41, as was the interaction,* F*(1, 154) = 7.01, *p* = .009, *η*^2^_p_ = .04, *d* = 0.43.
